# Interpretation of ANA Indirect Immunofluorescence Test Outside the Darkroom Using NOVA View Compared to Manual Microscopy

**DOI:** 10.1155/2014/149316

**Published:** 2014-02-24

**Authors:** Susan S. Copple, Troy D. Jaskowski, Rashelle Giles, Harry R. Hill

**Affiliations:** ^1^ARUP Laboratories, University of Utah School of Medicine, Salt Lake City, UT 84112, USA; ^2^Departments of Medicine, University of Utah School of Medicine, Salt Lake City, UT 84112, USA; ^3^INOVA Diagnostics, Inc. San Diego, 9900 Old Grove Road, CA 92131, USA; ^4^ARUP Institute for Clinical and Experimental Pathology, University of Utah School of Medicine, Salt Lake City, UT 84112, USA; ^5^Departments of Pathology, University of Utah School of Medicine, Salt Lake City, UT 84112, USA; ^6^Departments of Pediatrics, University of Utah School of Medicine, Salt Lake City, UT 84112, USA

## Abstract

*Objective.* To evaluate NOVA View with focus on reading archived images versus microscope based manual interpretation of ANA HEp-2 slides by an experienced, certified medical technologist. *Methods.* 369 well defined sera from: 44 rheumatoid arthritis, 50 systemic lupus erythematosus, 35 scleroderma, 19 Sjögren's syndrome, and 10 polymyositis patients as well as 99 healthy controls were examined. In addition, 12 defined sera from the Centers for Disease Control and 100 random patient sera sent to ARUP Laboratories for ANA HEp-2 IIF testing were included. Samples were read using the archived images on NOVA View and compared to results obtained from manual reading. *Results.* At a 1 : 40/1 : 80 dilution the resulting comparison demonstrated 94.8%/92.9% positive, 97.4%/97.4% negative, and 96.5%/96.2% total agreements between manual IIF and NOVA View archived images. Agreement of identifiable patterns between methods was 97%, with PCNA and mixed patterns undetermined. *Conclusion.* Excellent agreements were obtained between reading archived images on NOVA View and manually on a fluorescent microscope. In addition, workflow benefits were observed which need to be analyzed in future studies.

## 1. Introduction

The antinuclear antibody (ANA) test is a standard screening assay for detecting multiple antibodies that may be produced by a patient with an autoimmune or ANA associated rheumatic disease (AARD). Although there are several methodologies available to screen ANA, the American College of Rheumatology (ACR) issued a statement declaring HEp-2 indirect immunofluorescence (IIF) as the preferred method for ANA screening [1]. This declaration was based on the findings of a task force which investigated and collected information from physicians to evaluate nonstandardization of the various methodologies on the market for evaluating ANA. Using HEp-2 as a substrate allows the detection of more than 100 autoantibodies to different nuclear and cytoplasmic antigens [2]. These include antibodies to dsDNA, SS-A, Ro52, SS-B, RNP, centromere, Scl-70, Jo-1, ribosomal P, RNA Polymerase III, PM/Scl, Ku, Th/To, and Mi-2 to term the most important antigens. There are 5 to 6 indirect immunofluorescence (IIF) nuclear patterns that are commonly reported by most laboratories, namely, homogeneous, speckled, nucleolar, centromere, peripheral/rimmed, and proliferating cell nuclear antigen (PCNA). Laboratories performing the ANA IIF test typically report a positive result with a pattern and titer. This aids the physician when deciding what tests to order next, if any.

Performing IIF test is labor intensive, subjective, and prone to reader bias [3–7]. Many other variables affect the IIF test result such as the HEp-2 substrate, conjugate, microscope, type of bulb, and bulb life [2, 8–14]. Over the past few decades as newer technologies emerged for testing ANAs, there were fewer and fewer laboratorians with the knowledge and skill to perform ANA IIF testing. As a reference laboratory, ARUP continues to offer and perform HEp-2 IIF for ANA testing. Extensive time is required to train a technologist to be competent in reading and interpreting ANA IIF testing. In addition, there is a need for standardization and automation in ANA testing [1–3, 15].

Autoimmune laboratories have made strides in automation over the past decade but are still far behind other areas of the laboratory, such as chemistry with their fully automated instrumentation. Automated pattern interpretation of HEp-2 ANA was first described in 2002 by Perner et al. [16] Since then, there have been several studies of automated or digital IIF interpretation for positive and negative discrimination. Some systems incorporate pattern recognition algorithms. All conclude that automated IIF analysis will improve inter- and intralaboratory results [17–25]. The NOVA View instrument (INOVA Diagnostics, Inc., San Diego, CA) has been designed to address this need. NOVA View is an automated digital image analysis system, which is used for acquiring, analyzing, and interpreting ANA testing on HEp-2 cells, based on measured Light Intensity Units (LIU) and pattern recognition. NOVA View results are expressed in LIU and interpreted as negative or positive based on a preset cutoff. The cutoff intensity is preset by INOVA and may be adjusted for the customer based on their patient population and performance goals. The patented process produces three to five images per patient sample. The automated scan is followed by visual verification of the digital images, allowing for either confirmation or revision of results by the operator. NOVA View software recognizes five basic patterns: homogeneous, speckled, centromere, nucleolar, and nuclear dots. Pattern recognition is based on a software algorithm that analyzes the intensity and distribution of the fluorescent light over the area of the nuclei based on specific criteria. Mixed patterns may not be recognized by the software and may be reported as “unrecognized.” In these cases the final pattern is determined by the user during the revision and confirmation of the digital images.

Based on the recommendation of the ACR for the use of HEp-2 IIF to test for ANA, we aimed to compare the agreement of the NOVA View archived images to the interpretation of the same samples on a manual fluorescent microscope interpreted by a certified medical technologist, with emphasis on agreement of end point titer. In addition, the data were used to calculate ANA titers and positivity rate in various AARD.

## 2. Materials and Methods

### 2.1. Clinical Samples

Clinically defined serum samples from patients suffering from SLE (*n* = 50), rheumatoid arthritis (RA, *n* = 44), SSc (*n* = 35), Sjögren's syndrome (SjS, *n* = 19), and polymyositis (PM, *n* = 10) were included. Diagnoses were established as previously described or according to the respective disease classification criteria [26]. In addition, 99 healthy adult donor sera which consisted of 70% female and 30% male between the ages of 19 to 59 years of age were tested.

### 2.2. CDC ANA Reference Panel

International reference serum panel (CDC ANA #1–12) was obtained from the Centers of Disease Control and Prevention (CDC) (http://asc.dental.ufl.edu/ReferenceSera.html). (Biological Reference Reagents, NCID/SRP/BRR, Mailstop C-21, Centers for Disease Control and Prevention (CDC), 1600 Clifton Rd. N.E., Atlanta, GA, U.S.A).

### 2.3. Consecutive Routine Samples

Lastly, 100 consecutive samples from an individual client, sent to ARUP Laboratories for ANA IIF testing, were reviewed. All patient samples included in the study were deidentified according to the University of Utah Institutional Review Board-approved protocol number 7275 to meet the Health Information Portability and Accountability Act Patient Confidentiality Guidelines.

### 2.4. Microscopes and Indirect Immunofluorescence Reagents

NOVA Lite HEp-2 IgG ANA with DAPI kit and the NOVA View instrument with 1.0.2 software containing a cut-off value of 100 LIU for positive results (INOVA Diagnostics, San Diego, CA). The conjugate used in this assay contains the usual FITC fluorophor along with diamidino-2-phenylindole (DAPI), a blue nuclear stain that selectively binds to double stranded DNA. DAPI allows the instrument to “find” the cells at a 400 nm wavelength. If the cell density is insufficient or there are no cells in the well, the instrument will not switch to FITC but will produce an “*X*,” indicating an inadequate number or that no cells were found. Once the correct number of cells has been identified, the instrument switches to a 490 nm wavelength for FITC identification and quantification of antibody in the sample. NOVA View has 5 preselected fields where it collects a digital image producing 5 images on the screen when the sample is positive. These five preselected fields mimic the areas where a technologist would read with a manual microscope. If the sample is negative, three images are produced.

For the manual reading, a Nikon Eclipse 400 with an LED light source (ARUP Laboratories, Salt Lake City, Utah) was used.

All samples were processed manually and read on both the Nikon microscope and NOVA View, archived images with software version 1.0.2 by a board certified medical technologist. The technologist was blinded to sample classification and has 5 years of reading IIF daily at ARUP laboratories. Intensive training and continuous reading are needed for a technologist to accurately interpret HEp-2 ANA. At ARUP Laboratories, and other facilities, people who interpret HEp-2 ANA on clinical sera must be board certified. In order to read ANA IIF accurately and consistently they read daily and are challenged by internal and external surveys. Patterns recorded at ARUP include speckled, homogeneous, centromere, nucleolar, and nuclear dots, PCNA, and NuMA along with comments on cytoplasmic fluorescence observed.

## 3. Results

### 3.1. Agreement between Manual and NOVA View Interpretation

At a 1 : 40/1 : 80 dilution the resulting comparison demonstrated 94.8%/92.9% positive, 97.4%/97.4% negative, and 96.5%/96.2% total agreements (Tables 1 and 2). The majority of discrepant results between the manual and the archived based interpretation were ±1 dilution difference. The highest fluctuation between results was seen at the 1 : 40 dilution. A total of 13 samples that were called positive by one method of reading and negative by the other all had titers of 1 : 40 or 1 : 80 and <1 : 40 (Table 1). One sample demonstrated a PCNA pattern, a pattern which is not recognized by the NOVA View system. Therefore we aimed to evaluate if this pattern can be identified as PCNA pattern by the technologist reading the NOVA View archived images. The archived image of the PCNA pattern was clear and easy to interpret.

**Figure 1:**
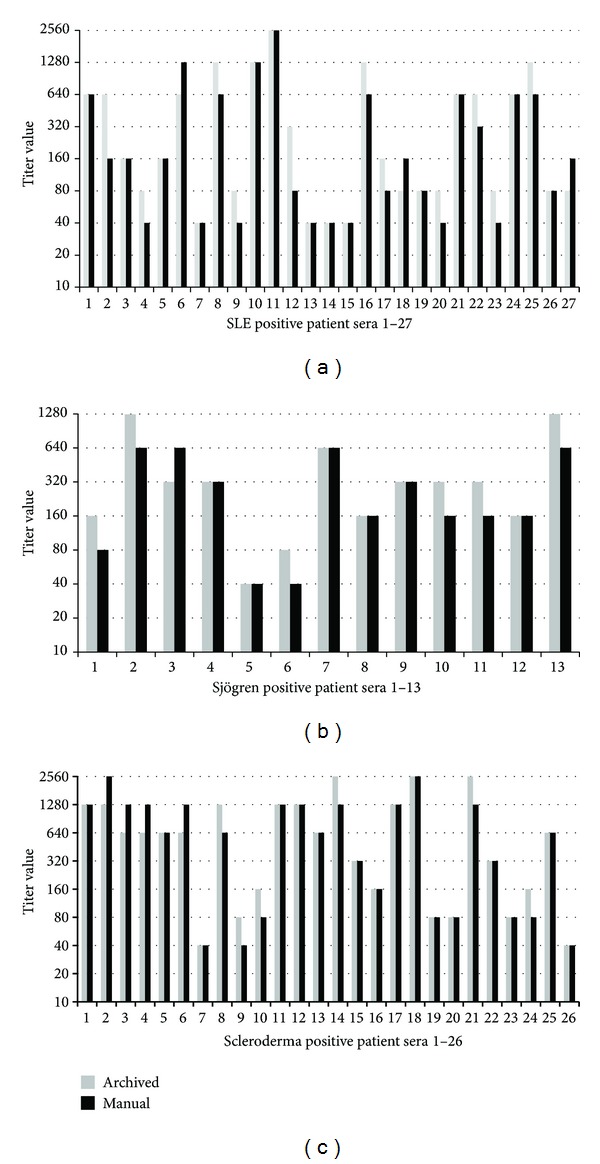
Antinuclear antibodies in different patient cohorts. (a) 27 positive systemic lupus erythematosus (SLE) patient sera titered at 1 : 40 through 1 : 2560. (b) Thirteen positive Sjögren's syndrome (SjS) patient sera titered at 1 : 40 through 1 : 1280. (c) 26 positive scleroderma patient sera titered at 1 : 40 through 1 : 2560. The gray bar represents the end point titer read on the NOVA View archived image. The black bar represents the titer read on a traditional manual microscope. All titers were read by the same technologist.

Of the 100 samples sent to ARUP Laboratories for routine ANA testing, 63% were negative and 37% were positive. Titers were within plus or minus a doubling dilution between the manual and the NOVA View archived image results, and ranged from 1 : 40 to 1 : 2560 (Figure 1). Patterns matched 100%.

**Figure 2:**
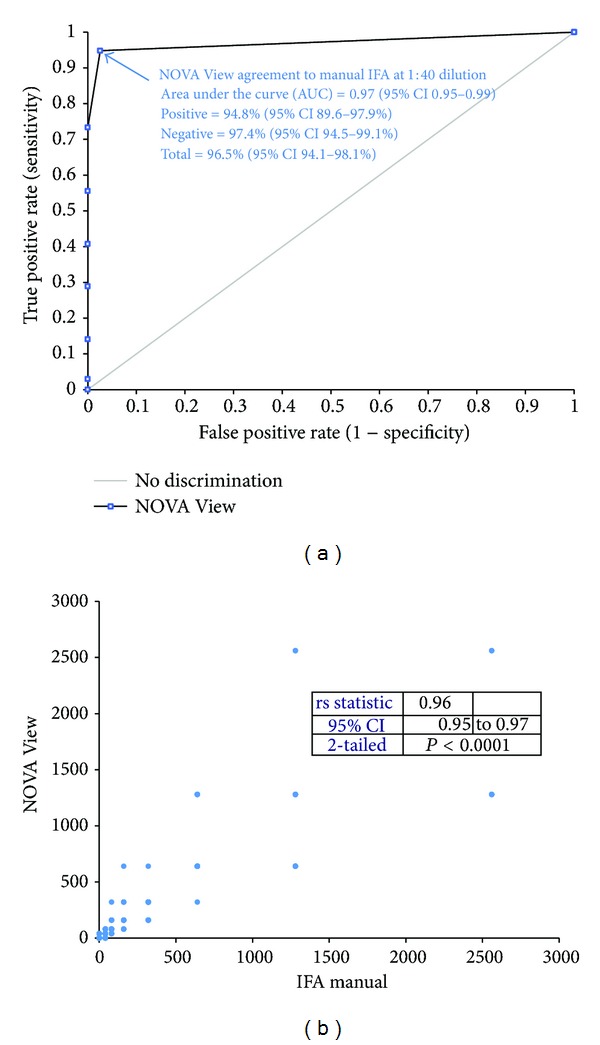
Agreement and correlation between manual and NOVA View archived image based reading. (a) Receiver operating characteristic (ROC) analysis. Results of the manual indirect immunofluorescence interpretation were grouped into positive and negative. Subsequently, the titers obtained from the NOVA View interpretation were used to generate a ROC curve showing very good agreement (*n* = 369). (b) Spearman's correlation (all samples, *n* = 369) between manual indirect immunofluorescence interpretation and NOVA View. Excellent correlation of rho = 0.96 was found.

Good agreement and correlation between manual and NOVA View archived based reading were found. Results of the manual IIF interpretation were grouped into positive and negative. Subsequently, the titers obtained from the NOVA View archived image based interpretation were used to generate a receiver operating characteristic (ROC) curve showing very good agreement (Figure 2(a)). Spearman's correlation (all samples, *n* = 369) between IIF interpretation and NOVA View showed excellent correlation of rho = 0.96 (Figure 2(b)).

### 3.2. CDC Samples

The 12 CDC samples produced excellent correlation for pattern and titer (data not shown). All samples with ANA were positive with titers ranging from 1 : 40 to 1 : 320. The patterns match their original description of the antibody specificity.

### 3.3. Clinically Defined Samples

**Figure 3:**
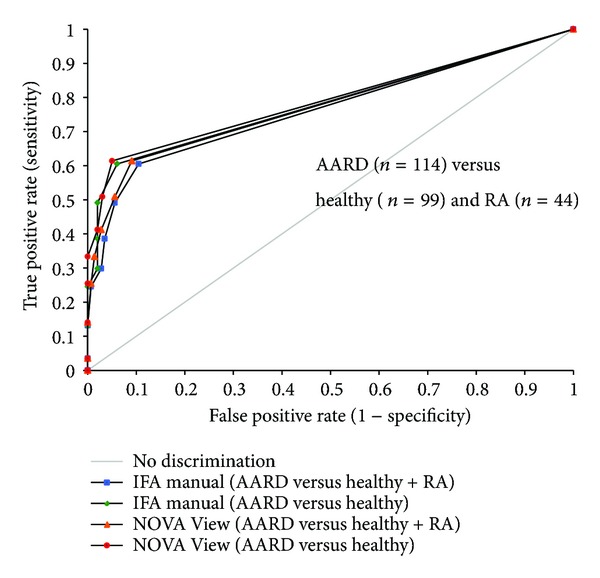
Comparative receiver operating characteristic (ROC) analyses. ROC for manual indirect immunofluorescence (IIF) manual interpretation and NOVA View results for ANA related autoimmune rheumatic disease (AARD) versus healthy controls and rheumatoid arthritis (RA). The ROC curves were similar for manual IIF and NOVA View, and as expected, specificity improves for both methods when RA patients are removed from analysis.

**Figure 4:**
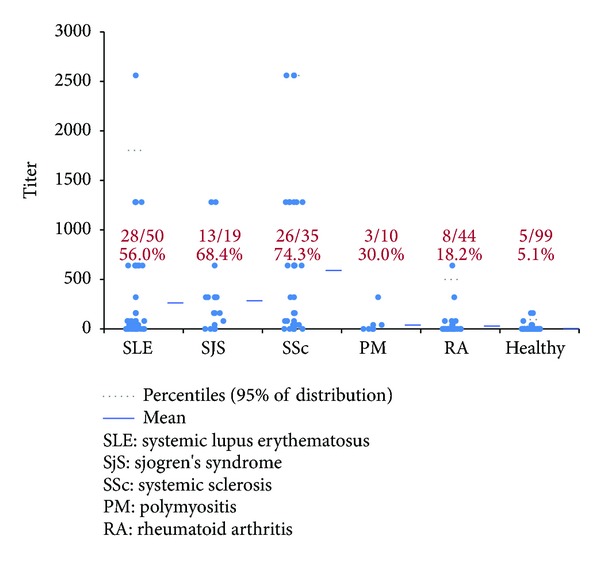
Antinuclear antibody titer and prevalence among ANA associated rheumatic diseases (AARD, *n* = 114), rheumatoid arthritis (RA, *n* = 44), and healthy controls (*n* = 99). Results are based on NOVA View archived images (similar results were found using manual reading).

The ROC analyses revealed similar discrimination between AARD and controls using the manual and the NOVA View archive reading (Table 3, Figure 3). A comparative descriptive analysis (Figure 4) demonstrated positivity in 56.0% of SLE, in 68.4% of SjS, in 74.3% of SSc, and in 30.0% of PM patients. In the control groups, 18.2% of the RA and 5.1% of the healthy individuals were ANA positive. Two of the 99 healthy donors demonstrated a result of 1 : 160 speckled pattern by manual microscopy and NOVA View archived image.

### 3.4. Workflow Analysis

Although not the focus of our study, we investigated the impact of the NOVA View instrument in the laboratory workflow. We found that the system is a walk-away platform, with user friendly software, and the ability to interface with bar coded slides for positive patient identification.

## 4. Discussion

The recommendation of the ACR to use IIF as the preferred method has triggered the development and validation of automated systems for ANA determination. Although the ANA IIF test is the recommended method for ANA testing, the method has significant limitations, including a high degree of subjectivity [27]. With the availability of novel digital imaging systems, this limitation can be overcome [27]. However, careful evaluation and validation of those systems are required to ensure that the ANA results do not sacrifice clinical accuracy. One of the systems, the NOVA View, was evaluated in our study. In the beta software version 1.0.2 of NOVA View we found the instrument produced high quality images and excellent agreement with manual IIF testing. Properly comparing the archived NOVA View results to the manual results and the negative and positive sera, along with titer outcomes, demonstrated 97% concordance, in this study.

The NOVA View has a walk-away platform, user friendly software, and the ability to interface with bar coded slides for positive patient identification. In addition, the patient images are stored for later viewing without fluorescent burnout. However, the impact on the workflow might vary from laboratory to laboratory and needs to be quantified in further studies. The good agreement between interpretation using a microscope and using archived images on a screen holds promise to avoid the dark room, which is a source of transcription errors of results.

The specificity against healthy individuals in this study was in keeping with recent recommendations for the determination of anticellular antibodies [28]. However, the prevalence in SLE patients was somewhat lower than expected. This might be explained by the SLE population used and the relatively small cohort.

Since we did not analyze the performance of the NOVA View in terms of positive/negative discrimination and pattern recognition, further studies are needed. In recent years, several of those studies have already been performed [17–23, 25, 27, 29]. The internal LIU cutoff value causes the instrument to display the term “negative” for a sample that produces less than 100 LIU, whereas a positive result is displayed if the LIU is 100 or greater. The preset LIU cutoff of 100 does not always correlate between instrument generated outcome and manual microscopy on low positive/negative samples. The LIU cutoff can be adjusted to closely match the laboratory's manual reading during the validation process if desired. This does not change the image produced by NOVA View. Among many other systems, the NOVA View is an automated image recognition instrument.

Since this study, NOVA View has had two software updates. The current version, 1.0.3.1, contains a Single Well Titer (SWT) application that utilizes the LIU and assigns pattern to produce a calculated titer from one well. A recent study by Schouwers et al. concluding the estimation of fluorescent intensity offers clinically useful information and value added reporting [29].

Further studies are desired to underline the clinical utility of the NOVA View system in diagnostic specimens.

## Figures and Tables

**Table 1:** NOVA View agreement with manual IIF: 1 : 40 screening dilution.

All patients (*n* = 369)	Manual IIF			Percent agreement (95% confidence)
	Positive	Negative	Total	
NOVA View				
Positive	128	6	134	Positive agreement = 94.8% (89.6–97.9%)
Negative	7	228	235	Negative agreement = 97.4% (94.5–99.1%)
Total	135	234	369	Total agreement = 96.5% (94.1–98.1%)
				*κ* = 0.92 (95% CI 0.88–0.96)

*N*: number of patients tested at a 1 : 40 dilution for HEp-2 ANA antibodies.

Manual results were read on a Nikon Eclipse 400 fluorescent microscope with an LED light source.

NOVA View confirmed results = archived images reviewed and confirmed by the operator.

**Table 2:** NOVA View agreement with manual IIF: 1 : 80 screening dilution.

All patients (*n* = 369)	Manual IIF			Percent agreement (95% confidence)
	Positive	Negative	Total	
NOVA View				
Positive	92	7	99	Positive agreement = 92.9% (86.0–97.1%)
Negative	7	263	270	Negative agreement = 97.4% (94.7–99.0%)
Total	99	270	369	Total agreement = 96.2% (93.7–97.9%)
				*κ* = 0.90 (95% CI 0.85–0.95)

*N*: number of patients tested at a 1 : 80 dilution for HEp-2 ANA antibodies.

Manual results were read on a Nikon Eclipse 400 fluorescent microscope with an LED light source.

NOVA View confirmed results = images reviewed and confirmed by the operator.

**Table 3:** Clinical sensitivity and specificity.

Assay	Sensitivity % (95% CI)	Specificity % (95% CI)	Excluding RA samples specificity % (95% CI)
Manual IIF, 1 : 40 dilution	60.5 (50.9–69.6)	89.5 (83.3–94.0)	93.9 (87.3–97.7)
NOVA View, 1 : 40 dilution	61.4 (51.8–70.4)	90.9 (85.0–95.1)	94.9 (88.6–98.3)
Manual IIF, 1 : 80 dilution	49.1 (39.6–58.7)	94.4 (89.3–97.6)	98.0 (92.9–99.8)
NOVA View, 1 : 80 dilution	50.9 (41.3–60.4)	94.4 (89.3–97.6)	97.0 (91.4–99.4)

## References

[1] Meroni PL, Schur PH (2010). ANA screening: an old test with new recommendations. *Annals of the Rheumatic Diseases*.

[2] Burlingame RW, Peebles C, Pollard KM (2006). Detection of antibodies. *Autoantibodies and Autoimmunity: Molecular Mechanisms in Health and Disease*.

[3] Copple SS, Giles SR, Jaskowski TD, Gardiner AE, Wilson AM, Hill HR (2012). Screening for IgG antinuclear autoantibodies by HEp-2 indirect fluorescent antibody assays and the need for standardization. *American Journal of Clinical Pathology*.

[4] Van BM, Van CC, Bossuyt X (2009). Current practices in antinuclear antibody testing: results from the belgian external quality assessment scheme. *Clinical Chemistry and Laboratory Medicine*.

[5] Fritzler MJ, Wiik A, Tan EM (2003). A critical evaluation of enzyme immunoassay kits for detection of antinuclear autoantibodies of defined specificities. III. comparative performance characteristics of academic and manufacturers’ laboratories. *Journal of Rheumatology*.

[6] Peterson LK, Wells D, Shaw L, Velez MG, Harbeck R, Dragone LL (2009). Novel method for quantitative ANA measurement using near-infrared imaging. *Journal of Immunological Methods*.

[7] Sack U, Conrad K, Csernok E (2009). Autoantibody detection using indirect immunofluorescence on HEp-2 cells. *Annals of the New York Academy of Sciences*.

[8] Egner W (2000). The use of laboratory tests in the diagnosis of SLE. *Journal of Clinical Pathology*.

[9] Fenger M, Wiik A, Hoier-Madsen M (2004). Detection of antinuclear antibodies by solid-phase immunoassays and immunofluorescence analysis. *Clinical Chemistry*.

[10] Bradwell AR, Hughes RG, Karim AR, Detrick B, Hamilton RG, Folds JD (2006). Immunofluorescent antinuclear antibody tests. *Manual of Clinical Laboratory Immunology*.

[11] Emlen W, O’neill L (1997). Clinical significance of antinuclear antibodies: comparison of detection with immunofluorescence and enzyme-linked immunosorbent assays. *Arthritis and Rheumatism*.

[12] Tonutti E, Bassetti D, Piazza A (2004). Diagnostic accuracy of ELISA methods as an alternative screening test to indirect immunofluorescence for the detection of antinuclear antibodies. Evaluation of five commercial kits. *Autoimmunity*.

[13] Tan EM, Smolen JS, Mcdougal JS (1999). A critical evaluation of enzyme immunoassays for detection of antinuclear autoantibodies of defined specificities. I. Precisionsensitivity, and specificity. *Arthritis & Rheumatology*.

[14] Swaak AJ (2000). Diagnostic significance of antinuclear antibodies in clinical practice. *Nederlands Tijdschrift Voor Geneeskunde*.

[15] Qin X, Tao X, Chen ZJ (2009). Comparison of indirect immunofluorescence assay and ELISA for detecting antinuclear antibodies and anti-double-stranded DNA antibodies. *Nan Fang Yi Ke Da Xue Xue Bao*.

[16] Perner P, Perner H, Müller B (2002). Mining knowledge for Hep-2 cell image classification. *Journal Artificial Intelligence in Medicine*.

[17] Egerer K, Roggenbuck D, Hiemann R (2010). Automated evaluation of autoantibodies on human epithelial-2 cells as an approach to standardize cell-based immunofluorescence tests. *Arthritis Research & Therapy*.

[18] Hiemann R, Buttner T, Krieger T, Roggenbuck D, Sack U, Conrad K (2009). Challenges of automated screening and differentiation of non-organ specific autoantibodies on HEp-2 cells. *Autoimmunity Reviews*.

[19] Willitzki A, Hiemann R, Peters V (2012). New platform technology for comprehensive serological diagnostics of autoimmune diseases. *Clinical and Developmental Immunology*.

[20] Voigt J, Krause C, Rohwader E (2012). Automated indirect immunofluorescence evaluation of antinuclear autoantibodies on HEp-2 cells. *Clinical and Developmental Immunology*.

[21] Roggenbuck D, Hiemann R, Schierack P, Reinhold D, Conrad K (2013). Digital immunofluorescence enables automated detection of antinuclear antibody endpoint titers avoiding serial dilution. *Clinical Chemistry and Laboratory Medicine*.

[22] Bonroy C, Verfaillie C, Smith V (2013). Automated indirect immunofluorescence antinuclear antibody analysis is a standardized alternative for visual microscope interpretation. *Clinical Chemistry and Laboratory Medicine*.

[23] Bossuyt X, Cooreman S, De BH (2013). Detection of antinuclear antibodies by automated indirect immunofluorescence analysis. *Clinica Chimica Acta*.

[24] Foggia P, Percannella G, Soda P, Vento M (2013). Benchmarking HEp-2 cells classification methods. *IEEE Transactions on Medical Imaging*.

[25] Roggenbuck D, Hiemann R, Bogdanos D, Reinhold D, Conrad K (2013). Standardization of automated interpretation of immunofluorescence tests. *Clinica Chimica Acta*.

[26] Mahler M, Fritzler MJ, Bluthner M (2005). Identification of a SmD3 epitope with a single symmetrical dimethylation of an arginine residue as a specific target of a subpopulation of anti-Sm antibodies. *Arthritis research & Therapy*.

[27] Bizzaro N, Antico A, Platzgummer S (2013). Automated antinuclear immunofluorescence antibody screening: a comparative study of six computer-aided diagnostic systems. *Autoimmunity Reviews*.

[28] Agmon-Levin N, Damoiseaux J, Kallenberg C (2013). International recommendations for the assessment of autoantibodies to cellular antigens referred to as anti-nuclear antibodies. *Annals of the Rheumatic Diseases*.

[29] Schouwers S, Bonnet M, Verschueren P (2013). Value-added reporting of antinuclear antibody testing by automated indirect immunofluorescence analysis. *Clinical Chemistry and Laboratory Medicine*.

